# Little agreement in GOLD category using CAT and mMRC in 450 primary care COPD patients in New Zealand

**DOI:** 10.1038/npjpcrm.2014.25

**Published:** 2014-07-24

**Authors:** Shaun Holt, Davitt Sheahan, Colin Helm, Chris Tofield, Andrew Corin, Janwillem W H Kocks

**Affiliations:** 1 School of Biological Sciences, Victoria University of Wellington, Wellington, New Zealand; 2 Papamoa Pines Surgery, Tauranga, New Zealand; 3 CentralMed, Tauranga, New Zealand; 4 Cameron Medical Clinic, Tauranga, New Zealand; 5 Department of General Practice, University Medical Center Groningen, University of Groningen, Groningen, The Netherlands; 6 GRIAC Research Institute, University Medical Center Groningen, University of Groningen, Groningen, The Netherlands; 7 Medical Research Institute of New Zealand, Wellington, New Zealand

The updated 2011 Global Initiative for Chronic Obstructive Lung Disease (GOLD) guidelines introduced a new method to categorise chronic obstructive pulmonary disease (COPD) patients according to risk and symptom level, whereas previously categorisation was based solely on lung function impairment.^1^ Risk is classified as either ‘low’ and ‘high’ based on lung function impairment (FEV_1_) and exacerbation history. Symptom level can be assessed by using the modified Medical Research Council (mMRC) dyspnoea scale, the COPD Assessment Test (CAT) or the Clinical COPD Questionnaire (CCQ), the latter being added in the 2013 update.^2^

The guidelines state the recommended first choice pharmacologic therapy, an alternative choice and other possible treatments for each category: A—‘low risk, less symptoms’; B—‘low risk, more symptoms’; C—‘high risk, low symptoms’; or D—‘high risk, more symptoms’.

It has been suggested that this new method of COPD patient classification should not be used in primary care because it could lead to inappropriate management recommendations and the categorisation process itself is too complex.^3^ As there are different methods to assess symptoms, with all three having different constructs and measurement properties,^4^ patients can fall into different categories according to the method used.^5^ As a result, clinicians can get different treatment recommendations depending on which method they use to assess symptoms.

We performed an audit of COPD patients in primary care to assess whether there were significant differences in GOLD category depending on whether the mMRC or the CAT were used, and also to determine the appropriateness of the medications that these patients were prescribed, according to GOLD recommendations.

Four hundred and fifty patients were assessed in three primary care practices in New Zealand, and data were collected on patient demographics, FEV_1_, exacerbation history, CAT and mMRC scores, and prescribed medication.

The prescribed medication level was rated as ‘undertreated’, ‘well treated’, ‘over treated’ or ‘other regime’. To establish each patient’s treatment level, their current medication use was compared with each GOLD treatment recommendation (first and second choice for GOLD stage A, B, C and D, thus resulting in eight ‘yes’ or ‘no’ according to, e.g., ‘second choice treatment for stage B’). ‘Well treated’ patients received pharmacotherapy that is listed for GOLD’s recommendation for first or second choice therapy for their category. Patients who were prescribed medication that corresponded to a lower or higher COPD category were rated ‘undertreated’ or ‘over treated’, respectively. Those who did not receive any medication were rated as ‘undertreated’ and those who were prescribed a combination of medications that did not correspond to any of the GOLD recommendations were rated as ‘other regime’. Cohen’s kappa, a statistical method designed to assess agreement over and above that due to chance between two raters who each classify cases into categories, was used to assess agreement of classifications of GOLD category and treatment levels. A kappa of 1 represents perfect agreement, whereas 0 indicates no agreement.

The mean age was 69 years (s.d.±10.1 years), 53% were males, 83% were New Zealand Europeans and 13% were Maori. The mean FEV_1_ was 56 (±18.5)% of predicted value.

A graphical summary of the findings is shown in Figure 1.

Classifying patients using mMRC resulted in 30, 16, 16 and 38% in groups A, B, C and D, and using CAT resulted in 17, 29, 8 and 46%, respectively. The agreement between the categorisation using mMRC or CAT according to Cohen’s kappa was 0.62.

More than 50% of patients included in this audit were prescribed medications that were not consistent with any first or second choice pharmacologic therapy listed in the GOLD guideline. A key finding was that 20% of patients were undertreated. In particular, when using the CAT, a large proportion in group B were undertreated, implying that a substantial proportion of patients with high levels of symptoms were not prescribed long-acting bronchodilators. In contrast, 68% of CAT-classified GOLD C patients received inhaled corticosteroids, which is not recommended for GOLD C patients. On average, patients in GOLD D used 2.7 (s.d. 1.2) different inhalers compared with 1.5 (s.d. 1.3) in GOLD A.

The agreement in treatment level between patients categorised using mMRC or CAT was 0.94.

The moderate agreement between using mMRC or CAT is similar to that seen in two other studies that had values of 0.51 (ref. 5) and 0.63 (ref. 6). In those studies and this audit, using the CAT led to a shift towards the ‘more symptoms’ categories, B and D. The likely reason for this is that the mMRC assesses only dyspnoea during exercise, whereas the CAT also assesses other parameters such as cough, phlegm, chest tightness, breathlessness going up hills/stairs, activity limitation, sleep, energy and confidence leaving home. Therefore, the CAT is more likely to reflect effects of COPD on a patient’s life than the mMRC. Also, the CAT might be more sensitive to effects of co-morbidity than the mMRC, e.g., orthopnea caused by heart failure might also affect sleep.

In particular, use of the CAT resulted in the recategorisation of a substantial number of patients from group A to B. This results in more patients having long-acting bronchodilators recommended for their treatment, and in this audit, it meant that the use of CAT meant that more patients were classified as undertreated in group B. The risk versus benefit of earlier treatment with long-acting bronchodilators is currently uncertain.

This audit and other similar studies are limited in that they have only assessed cross-sectional differences in GOLD categorisation and not changes in categorisation over time. The previous GOLD classification was solely based on FEV_1_ and as treatment did not greatly affect FEV_1_, there was only limited movement between categories and therefore treatment recommendations. However, data from primary care suggest that many people change categories over time,^7,8^ which could lead to step down of treatments. More studies are required to assess the effects of stepping down on outcomes. Although COPD is considered a progressive disease, a reduction in treatment could positively affect health by, e.g., reducing troublesome side effects. Also the medication(s) may have been initiated for the first, and perhaps, only exacerbation the patient will experience and therefore may not be required.

The decision as to which questionnaire to use in clinical practice and how this would affect outcomes could not be answered based on this study. To answer this question, a prospective longitudinal study in which treatments are administered strictly according to the categorisation based on the two (and now three) questionnaires recommended by GOLD and assessing important clinical outcomes would be needed. However, based on feasibility and applicability for primary care, the International Primary Care Respiratory Group has previously recommended the use of the CCQ or CAT.^9^

In summary, this audit found that there was a marked difference in GOLD category depending on the method of symptom assessment and that many patients are undertreated or prescribed a treatment regime not consistent with the GOLD guideline.

## Figures and Tables

**Figure 1:**
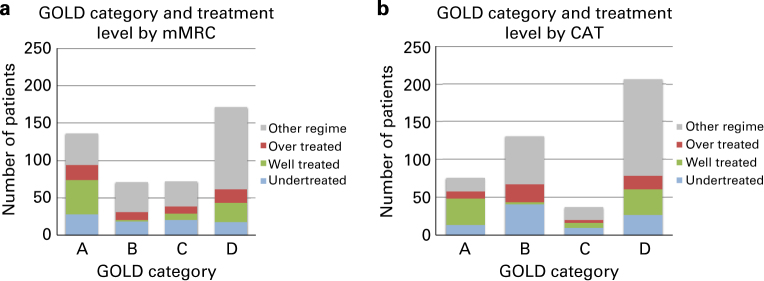
Global Initiative for Chronic Obstructive Lung Disease (GOLD) category by treatment level by (**a**) modified Medical Research Council Dyspnoea Scale and (**b**) COPD Assessment Test (CAT).

## Notes

The authors declare that they have no conflicts of interest in relation to this article. JWHK is an Associate Editor of *npjPCRM*, but was not involved in the editorial review of, nor the decision to publish, this article. GlaxoSmithKline developed the CAT and holds the CAT’s copyrights.
